# Improved chemical fixation of lipid-secreting plant cells for transmission electron microscopy

**DOI:** 10.1093/jmicro/dfac018

**Published:** 2022-04-05

**Authors:** Shingo Kiyoto, Takuji Ichino, Tatsuya Awano, Kazufumi Yazaki

**Affiliations:** Laboratory of Tree Cell Biology, Division of Forest and Biomaterials Science, Graduate School of Agriculture, Kyoto University, Sakyo-ku, Kyoto 606-8502, Japan; Laboratory of Plant Gene Expression, Research Institute for Sustainable Humanosphere (RISH), Kyoto University, Uji, 611-0011, Japan; Laboratory of Tree Cell Biology, Division of Forest and Biomaterials Science, Graduate School of Agriculture, Kyoto University, Sakyo-ku, Kyoto 606-8502, Japan; Laboratory of Plant Gene Expression, Research Institute for Sustainable Humanosphere (RISH), Kyoto University, Uji, 611-0011, Japan

**Keywords:** imidazole, *Lithospermum erythrorhizon*, malachite green, *p*-phenylenediamine, shikonin, transmission electron microscopy

## Abstract

Cultured *Lithospermum erythrorhizon* cells were fixed with a new fixation method to visualize the metabolism of shikonin derivatives, the lipophilic naphthoquinone pigments in Boraginaceae. The new fixation method combined glutaraldehyde containing malachite green, imidazole–osmium and *p*-phenylenediamine treatments, and cells were then observed with a transmission electron microscope. The method prevented the extraction of lipids, including shikonin derivatives, and improved the visualization of subcellular structures, especially the membrane system, when compared with that of conventional fixation. The improved quality of the transmission electron micrographs is because malachite green ionically binds to the plasma membrane, organelles and lipids and acts as a mordant for electron staining with osmium tetroxide. Imidazole promotes the reaction of osmium tetroxide, leading to enhanced electron staining. *p*-Phenylenediamine reduces osmium tetroxide bound to cellular materials and increases the electron density. This protocol requires only three additional reagents over conventional chemical fixation using glutaraldehyde and osmium tetroxide.

## Introduction

**Fig. 1.**
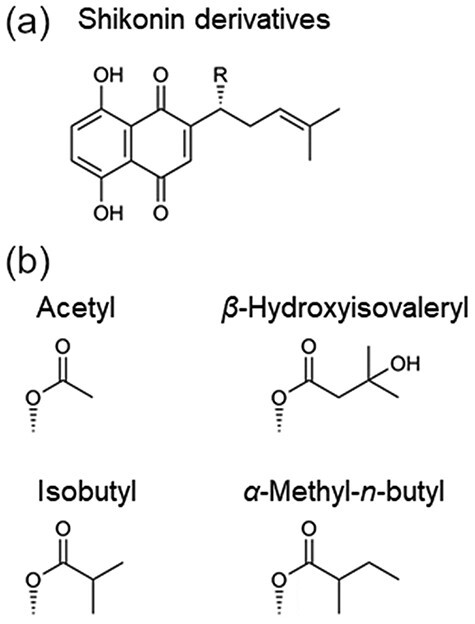
Shikonin derivatives produced by *L. erythrorhizon*. (a) Structures of shikonin derivatives. (b) Chemical prefixes of the shikonin derivatives and corresponding structures of R in (a).

Plants produce many lipophilic compounds to endure stresses caused by infection, dryness, UV irradiation, mechanical stress and temperature variation. These metabolites often provide benefits to human health as natural medicines [1]. For example, *Lithospermum erythrorhizon* Sieb. et Zucc. (Boraginaceae) secretes shikonin derivatives (Fig. 1) from epidermal cells and accumulates these derivatives in the root bark. Shikonin derivatives, lipophilic red naphthoquinone pigments, have medicinal effects such as anticancer, anti-inflammatory, wound-healing and other various beneficial properties [2,3]. The plant is a good model for investigating subcellular structures associated with lipid metabolism because the cultivation methods for callus and hairy roots are well established [4,5], and the production of shikonin derivatives can be controlled easily by the shikonin-producing M9 medium [6]. Several transmission electron microscopy (TEM) observations have been performed to investigate production patterns of shikonin derivatives in *L. erythrorhizon* cells [7,8]. However, the fixation of lipophilic compounds in these cells was not consistent. For example, high electron-dense lipid droplets were observed inside cultured cells by Tsukada and Tabata [7] but not in hairy roots of this plant by Tatsumi *et al*. [8], even though the specimens in both research efforts were fixed with conventional chemical fixation. This variability in TEM observations arises because slight differences in experimental conditions affect the preservation of lipid droplets. Thus, a reliable fixation method to visualize lipids is needed.

TEM observation of living tissue requires a fixation process to prevent autolysis, decay, shrinkage and displacement. The conventional chemical fixation method involves prefixation with glutaraldehyde and postfixation with osmium tetroxide. Glutaraldehyde and osmium tetroxide act as crosslinking reagents of amino groups and carbon double bonds, respectively [9,10]. Osmium tetroxide also reacts with several kinds of amino acids [11] and contributes to the electron staining of cellular components because of the high electron density of osmium. However, conventional chemical fixation has several problems that cause artifacts. First, considerable amounts of phospholipids are removed during glutaraldehyde fixation [12,13]. Second, osmium staining diffuses in an aqueous system [14]. Third, these fixative reagents, especially osmium tetroxide, require an extended period to penetrate tissues [15,16], and deformation can occur during the fixation process. These problems can distort the cell morphology and may lead to incorrect interpretation.

Freeze substitution after rapid freezing or high-pressure freezing is a method to observe cellular structures without deformation. This method immobilizes cells instantly by freezing, and fixative reagents dissolved in organic solvents penetrate the frozen tissues without deforming cellular structures. However, this method requires an expensive freezing device and cooling medium and limits the size of the sample examined. In addition, organic solvents access samples before cross-linkage formation by fixative reagents in freeze substitution. This is a critical disadvantage in observing subcellular structures containing lipophilic metabolites. Thus, an improved chemical fixation method is required to observe lipophilic metabolites in their near-native state.

Several fixative reagents for observation of lipid droplets have been reported. Teichman *et al*. [17] reported that glutaraldehyde containing malachite green fixes highly osmiophilic materials lost during conventional fixation. Angermüller and Fahimi [14] reported that imidazole-buffered osmium tetroxide strongly stains and insolubilizes lipid droplets. Ledingham and Simpson [18] reported that *p*-phenylenediamine treatment after postfixation enhances electron staining of membrane systems and lipid droplets. However, only a few studies have been conducted on lipid droplets in plant tissues by using these methods [19–23].

In this study, we applied a combination of these lipid fixation methods to fix shikonin derivatives in cultured *L. erythrorhizon* cells. The results show that the new fixation method prevents the extraction of lipids, including shikonin derivatives. In addition, the method further improves the visualization of subcellular structures by TEM. The new fixation method requires only a few additional steps when compared with that of conventional chemical fixation, and the cost is not much higher either.

## Materials and methods

### Plant materials

As a model of lipid-producing cells, cultured *L. erythrorhizon* cells were used. This plant secretes shikonin derivatives, which are highly hydrophobic red naphthoquinone pigments, from epidermal cells and accumulates these derivatives mainly in the root bark. A high shikonin-producing cell culture line (Mp) was initially established by Yamamura *et al*. [24]. This cell line was further selected to obtain and establish a newly cultured cell line (Mpr). The cultured cells (Mpr) were maintained in Linsmaier and Skoog (LS) medium [25], where shikonin derivatives are not produced. For inducing shikonin production, those white cells were transferred into M9 medium [6]. After inoculation in M9 medium, the cells were cultured on a rotary shaker (100 rpm) at 23°C for 20 days in the dark to produce shikonin derivatives. Cell suspension cultures turned deep red [3,26]. As a negative control for shikonin production, nonpigmented cells in LS medium were also prepared.

### Fixation and embedding

Some of the cultured cells were fixed by a conventional fixation method as follows. The cells were fixed with 5% (v/v) glutaraldehyde in M9 medium for 2 h at room temperature and then overnight at 4°C (prefixation). The sample was washed three times with M9 medium and three times with 50 mM Na-piperazine-*N*, *N*′-bis (2-ethane sulfonic acid; Na-PIPES) buffer (pH 6.8) for 10 min each. Cells were then fixed in 2% (w/v) osmium tetroxide in buffer for 2 h at room temperature (postfixation). After repeatedly washing with deionized water, samples were dehydrated in graded ethanol (30%, 50%, 70%, 90%, 10 min each, 100% 3 × 10 min). After dehydration, samples were embedded using the Spurr Low Viscosity Embedding kit (Polysciences Inc., Warrington, PA, USA) and according to the manufacturer’s instructions.

One of the following modifications was applied to the conventional fixation process to fix lipophilic compounds such as shikonin derivatives: (i) Prefixation reagent was replaced with 3% (v/v) glutaraldehyde and 0.1% (w/v) malachite green oxalate in 50 mM Na-PIPES buffer (pH 6.8). After prefixation, the sample was washed six times with the buffer. (ii) Postfixation reagent was replaced with 1% (w/v) osmium tetroxide and 80 mM imidazole in the buffer, and the reaction time was reduced to 30 min. (iii) Upon reaching the 70% ethanol dehydration step, samples were treated with 1% (w/v) *p*-phenylenediamine in 70% ethanol for 30 min. These fixation methods were termed the malachite green method, imidazole-osmium method and *p*-phenylenediamine method, respectively.

To further improve fixation effects, all of these modifications were applied to the fixation of some samples as follows: The cells were prefixed with 3% (v/v) glutaraldehyde and 0.1% (w/v) malachite green oxalate in 50 mM Na-PIPES buffer (pH 6.8) for 2 h at room temperature and overnight at 4°C. The sample was washed five times with the buffer for 10 min each. Cells were then fixed with 1% (w/v) osmium tetroxide and 80 mM imidazole-HCl buffer in 50 mM Na-PIPES buffer for 30 min at room temperature. Note that this postfixation reagent should be prepared just before the use as follows: One volume of 4% (w/v) osmium tetroxide aqueous solution (stock solution) and two volumes of 100 mM Na-PIPES buffer (pH 6.8) were mixed in a glass vial, followed by the addition of one volume of 320 mM imidazole-HCl buffer (pH 7.0). After postfixation, the sample was washed once with 80-mM imidazole-HCl in 50 mM Na-PIPES buffer (pH 6.8), twice with the Na-PIPES buffer and three times with deionized water for 10 min each. After washing, samples were dehydrated in graded ethanol (30%, 50%, 10 min each; 70% containing 1% (w/v) *p*-phenylenediamine, 30 min; 70%, 90%, 10 min each, 100% 3 × 10 min). After dehydration, samples were embedded using the Spurr Low Viscosity Embedding kit and according to the manufacturer’s instructions. This fixation method was named after the initial letters of the added reagents, i.e. the MGIP method.

### Light microscopy

Semithin sections (about 0.5 μm thick) were cut with an ultramicrotome from the samples fixed by the MGIP method. The sections were mounted in EUKITT® neo (O. Kindler & ORSAtec, Bobingen, Germany). The sections were observed under a light microscope (BX50, Olympus, Tokyo, Japan) equipped with a digital camera (DP72, Olympus).

### Transmission electron microscopy

Ultrathin sections (about 80 nm thick) were cut with an ultramicrotome and mounted on copper grids. The sections were stained with a 2% (w/v) aqueous solution of uranyl acetate for 30 min at room temperature, washed with deionized water and stained with Reynolds’ lead citrate [27] for 3 min. Sections were washed with distilled water, dried and observed under a TEM (JEM1400, JEOL, Tokyo, Japan) at an accelerating voltage of 80 kV. Some sections were also observed without poststaining or 15-min staining with an aqueous solution of 1% (w/v) potassium permanganate and 0.1% (w/v) sodium citrate.

### Spot tests

The spot test was performed on oleic acid, triolein and palmitic acid to verify the lipid fixation ability of the MGIP method. Ethanolic solutions of 10% (w/v) oleic acid, 1% (w/v) triolein and 1% (w/v) palmitic acid were prepared. An aliquot of these solutions (0.5 μl) was spotted on a filter paper. The spotting was repeated 10 times for triolein and palmitic acid. Three pieces of the filter papers were fixed by the imidazole–osmium method, a combination of the imidazole–osmium method and *p*-phenylenediamine method, or the MGIP method. After fixation, the pieces of the filter paper were immersed in a graded series of the Spurr Low Viscosity Embedding kit, placed on a glass slide and cured at 70℃. The filter paper pieces were observed under a stereomicroscope (SZX7, Olympus) equipped with a digital camera (DP22, Olympus). Several pieces of the filter paper were fixed by the MGIP method, embedded using the Spurr Low Viscosity Embedding kit and observed by light microscopy, as mentioned above.

## Results

### Light microscopy

**Fig. 2.**
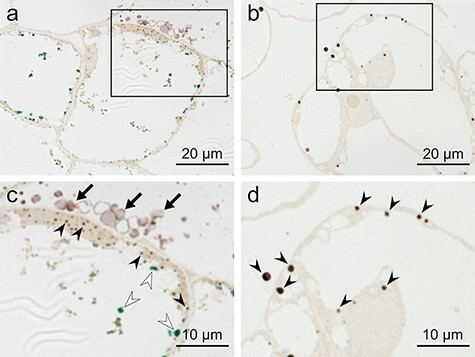
Light micrographs of *L. erythrorhizon* cultured cells fixed with the MGIP method. The sections were observed without post-embedded staining. (a) Cells cultured in M9 medium producing shikonin derivatives. (b) Cells cultured in LS medium as the nonproducing control. (c) Magnified image of the boxed region in (a). Solid and open arrowheads indicate lipid droplets in cytoplasm and materials in vacuole, respectively. Arrows indicate shikonin granules secreted to the extracellular spaces. (d) Magnified image of the boxed region in (b). Arrowheads indicate lipid droplets.

For cells cultured in M9 medium and fixed using the MGIP method, the cytoplasm and shikonin granules secreted into extracellular spaces (Fig. 2c, arrows) were stained orange, and lipid droplets in cytoplasm and materials in vacuole were stained black and green, respectively (Fig. 2c, solid and open arrowheads). There were fewer lipid droplets in cells cultured in LS medium, and lipids in the cells and extracellular spaces were stained black (Fig. 2d, arrowheads).

### Transmission electron microscopy

**Fig. 3.**
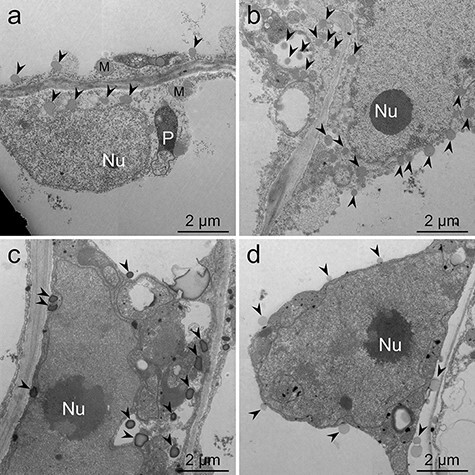
TEM images of *L. erythrorhizon* cultured cells producing shikonin derivatives. (a) Fixed with conventional fixation. (b) Fixed with the malachite green method. (c) Fixed with the imidazole–osmium method. (d) Fixed with the *p*-phenylenediamine method. The sections were stained with uranyl acetate and lead citrate. Arrowheads indicate lipid droplets. M, mitochondria; Nu, nucleolus; P, plastid.

In conventional fixation, only a few lipid droplets were observed in the cultured cells, and the electron density of lipid droplets, organelles and membrane systems was very poor (Fig. 3a, Supplementary Fig. S1). In the malachite green method, lipid droplets were preserved better than those observed using conventional fixation (Fig. 3b, Supplementary Fig. S2). In the imidazole–osmium method, lipid droplets were well preserved and showed high electron density although they had an electron-lucent central core (Fig. 3c, arrowheads, Supplementary Fig. S3). In the *p*-phenylenediamine method, improvement of electron density and preservation of lipid droplets were hardly observed when compared with that of conventional fixation (Fig. 3d, Supplementary Fig. S4).

**Fig. 4.**
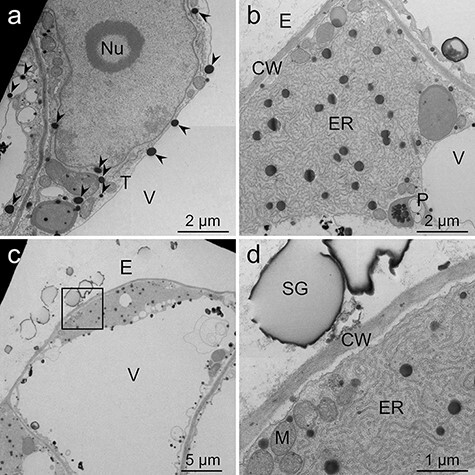
TEM images of *L. erythrorhizon* cultured cells producing shikonin derivatives fixed with the MGIP method. The sections were stained with uranyl acetate and lead citrate. (a) Nuclear membrane, organelles, lipid droplets and tonoplasts are clearly visible. Arrowheads indicate lipid droplets. (b) A highly developed ER and a lipid-storing plastid were observed. (c) A cell secreting shikonin derivatives. (d) Magnified image of the boxed region in (c). CW, cell wall; E, extracellular spaces; ER, endoplasmic reticulum; M, mitochondria; Nu, nucleolus; P, plastid; SG, shikonin granules secreted to extracellular spaces; T, tonoplast; V, vacuole.

**Fig. 5.**
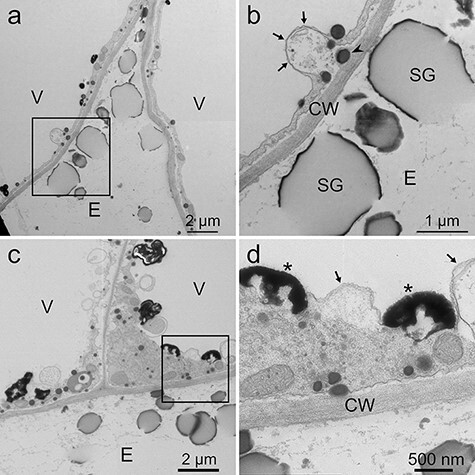
TEM images of *L. erythrorhizon* cultured cells producing shikonin derivatives fixed using the MGIP method. The sections were stained with uranyl acetate and lead citrate. (a, c) Cells secreting shikonin derivatives. (b) Magnified image of the boxed region in (a). Arrows indicate the folded plasma membrane and tonoplast. An arrowhead indicates a lipid droplet on the outer side of the folded plasma membrane. (d) Magnified image of the boxed region in (c). Arrows indicate a folded tonoplast. Asterisks indicate high-electron-dense materials attached to tonoplasts. CW, cell wall; E, extracellular spaces; SG, shikonin granules secreted to extracellular spaces; V, vacuole.

**Fig. 6.**
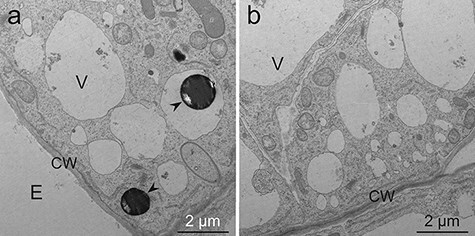
TEM images of *L. erythrorhizon* cultured cells not producing shikonin derivatives fixed with the MGIP method. The sections were stained with uranyl acetate and lead citrate. (a) A cell adjacent to the extracellular spaces. (b) A cell that is not adjacent to the extracellular spaces. Arrowheads indicate lipid droplets. CW, cell wall; E, extracellular spaces; V, vacuole.

In the MGIP method, lipid droplets showed high electron density (Fig. 4a, arrowheads). Subcellular structures were also clearly visible and distinguishable. For example, tonoplasts, lipid-storing plastids and mitochondria were clearly observed (Fig. 4a, b and d). In addition, shikonin granules secreted into the extracellular spaces were sometimes observed in cells cultured in M9 medium although they were sometimes cracked (Fig. 4d). The outer surface of the granules showed a higher electron density than the inner part. In cells cultured in M9 medium, highly developed endoplasmic reticulum (ER) and intense undulation of plasma membranes were often observed (Fig. 4b–d). Moreover, cells cultured in M9 medium were occasionally observed to have folded plasma membranes and tonoplasts (Fig. 5b and d, arrows). Lipid droplets were sometimes observed on the outer side of the folded plasma membrane (Fig. 5b, arrowhead). In addition, electron-dense materials are sometimes attached to tonoplasts (Fig. 5d, asterisks). In cells cultured in LS medium, high-electron-dense lipid droplets were observed at an even lower frequency, and undulation of plasma membranes was less intense when compared with that of cells cultured in M9 medium (Fig. 6a and b).

**Fig. 7.**
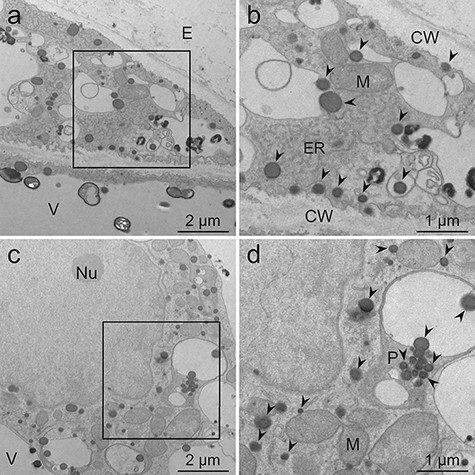
TEM images of *L. erythrorhizon* cultured cells producing shikonin derivatives fixed with the MGIP method. The sections were observed without post-embedded staining. (a) A cell adjacent to the extracellular spaces. (b) Magnified image of the boxed region in (a). Mitochondria, ER, lipid droplets and tonoplasts are visible. (c) The cytoplasm containing the nucleus and several organelles. (d) Magnified image of the boxed region in (c). A nuclear membrane and a lipid-storing plastid are visible. Arrowheads indicate lipid droplets. CW, cell wall; E, extracellular spaces; ER, endoplasmic reticulum; M, mitochondria; Nu, nucleolus; P, plastid; V, vacuole.

**Fig. 8.**
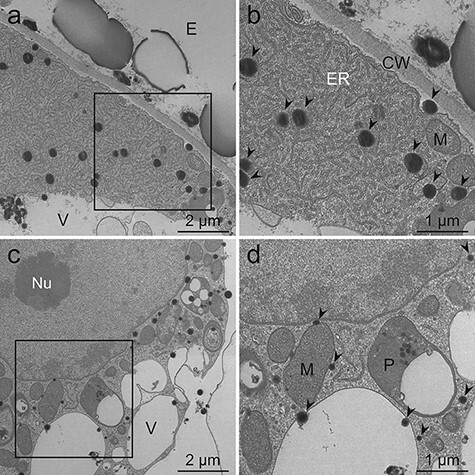
TEM images of *L. erythrorhizon* cultured cells producing shikonin derivatives fixed with the MGIP method. The sections were stained with potassium permanganate. (a) A cell adjacent to the extracellular spaces. (b) Magnified image of the boxed region in (a). Mitochondria, ER, lipid droplets and tonoplasts are visible. (c) The cytoplasm containing the nucleus and several organelles. (d) Magnified image of the boxed region in (c). A nuclear membrane and a lipid-storing plastid are clearly visible. Arrowheads indicate lipid droplets. CW, cell wall; E, extracellular spaces; ER, endoplasmic reticulum; M, mitochondria; Nu, nucleolus; P, plastid; V, vacuole.

Although post-embedded staining was omitted, the visibility of lipid droplets and subcellular structures was higher when using the MGIP method (Fig. 7) versus other methods with double staining using uranyl acetate and lead citrate (Fig. 3). Potassium permanganate staining improved the visualization of lipid droplets, organelles and the cell wall of cells fixed with the MGIP method (Fig. 8). Organelles such as the ER and the matrix in mitochondria were more evident in potassium permanganate-stained samples (Fig. 8c and d) than in samples stained with uranyl acetate and lead citrate (Fig. 4a, b and d).

### Spot tests

**Fig. 9.**
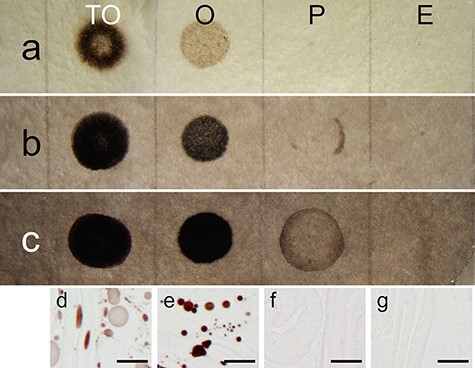
Spot tests on specific lipid standards. Stereoscopic micrographs of filter paper fixed with (a) the imidazole–osmium method, (b) a combination of the imidazole–osmium and *p*-phenylenediamine methods and (c) the MGIP method. Light micrographs of filter paper where (d) triolein, (e) oleic acid, (f) palmitic acid and (g) ethanol only, as a negative control, were spotted and fixed with the MGIP method. Scale bars indicate 10 μm. E, ethanol only, as a negative control; O, oleic acid; P, palmitic acid; TO, triolein.

In the imidazole–osmium method, triolein was stained black, and oleic acid was stained light orange, whereas the staining of palmitic acid was barely visible (Fig. 9a). In the combined imidazole–osmium and *p*-phenylenediamine method, both triolein and oleic acid were stained black (Fig. 9b). In the MGIP method, triolein and oleic acid were stained black, and palmitic acid was stained gray (Fig. 9c). In light microscopy, however, triolein and oleic acid were clearly observed, whereas palmitic acid was not observed, which is similar to the observation made in the negative control (Fig. 9d–g).

## Discussion

### Effects and mechanisms of fixation methods

A considerable proportion of phospholipids, which are the main components of the membrane, are extracted during glutaraldehyde fixation [12,13]. In addition, Angermüller and Fahimi [14] reported that osmium tetroxide stains lipid droplets very weakly and diffuses without imidazole. These reports explain the ambiguous appearance of subcellular structures when conventional fixation is used (Fig. 3a, Supplementary Fig. S1).

Glutaraldehyde solution containing malachite green was reported to fix phospholipids typically extracted during conventional fixation [13]. In addition, it was reported that malachite green also binds to cerebrosides, cholesterol, fatty acids, fatty aldehydes, glyceryl tripalmitate and β-lipoprotein [28]. In the cells cultured in M9 medium, malachite green stained materials in vacuole green (Fig. 2a and c). Teichman *et al*. [17] argued that ionic attraction between tertiary amine groups of malachite green and basophilic residues of the malachite-green-affinity material is followed by the formation of coordinate linkage between osmium tetroxide and malachite green. These reports indicate that malachite green binds to phospholipid membranes and lipophilic metabolites and acts as a mordant for electron staining with osmium tetroxide. This postulate explains the better preservation of lipids when using the malachite green method over conventional fixation (Fig. 3b, Supplementary Fig. S2).

In the imidazole–osmium method, electron staining of lipid droplets and subcellular structures was even stronger when compared with that of conventional fixation, despite the lower concentration of osmium tetroxide and shorter reaction time (Fig. 3c, Supplementary Fig. S3). Cleare *et al*. [29] suggested that nitrogen-donor ligands accelerate the reaction of alkenes with osmium tetroxide. Emerman and Behrman [30] reported that tertiary amine ligands promote the cross-linking reaction of proteins by osmium tetroxide. Angermüller and Fahimi [14] discussed that imidazole penetrates tissues and the plasma membrane because of its tertiary amine group, which is a common structure of local anesthetic reagents [31]. These reports explain the more rapid and intense reaction of osmium tetroxide in the imidazole–osmium method than in conventional fixation. Angermüller and Fahimi [14] also reported that imidazole prevents the diffusion of osmium staining. Therefore, electron staining by osmium tetroxide in the presence of imidazole may show closer to native locations of the osmiophilic compounds. However, several lipid droplets had electron-lucent central cores (Fig. 3c, arrowheads, Supplementary Fig. S3). This phenomenon is explained by lipid extraction during double staining with uranyl acetate and lead citrate [32].

Ledingham and Simpson [18] discussed that *p*-phenylenediamine treatment causes a reduction of osmium tetroxide bound to cellular materials and increases electron density. Boshier *et al*. [33] discussed that *p*-phenylenediamine protects stained lipids from extraction during dehydration. Despite these reports, increasing electron density and preservation of lipid droplets were hardly observed in the *p*-phenylenediamine method alone when compared with that of conventional fixation (Fig. 3d, Supplementary Fig. S4). However, *p*-phenylenediamine visualized osmium-stained components such as the cytoplasm and lipids by light microscopy (Fig. 2), as reported previously [34]. In addition, Nakao *et al*. [35] reported that treatment with a mixture of imidazole and *p*-phenylenediamine after postfixation with osmium tetroxide provides better electron staining of lipid droplets than treatment with imidazole or *p*-phenylenediamine alone. Their report indicates that *p*-phenylenediamine enhances the electron density of osmium in synergy with imidazole. This insight is consistent with the results of the spot test. The combination of the imidazole–osmium and *p*-phenylenediamine methods showed darker staining of unsaturated lipids than the imidazole–osmium method (Fig. 9a and b). Osmium appears black in reduced states [36], yielding higher electron density [37].

As mentioned above, these methods have different fixation mechanisms. Insights from previous reports investigated the idea that combining the three methods should improve the visualization of lipids and cellular structures more than each method alone. In the MGIP method, lipid droplets showed higher electron density, and the visibility of organelles was improved when compared with the results from the other fixation methods (Figs. 4–8). In addition, electron-lucent central cores in lipid droplets, which were sometimes observed by the imidazole–osmium method, were not observed by the MGIP method. This observation indicates that lipid droplets were insoluble to uranyl acetate and lead citrate when using the MGIP method (Fig. 4). The probable mechanism of improved visualization can be explained as follows. Malachite green ionically binds to materials in tissues and acts as a mordant for osmium tetroxide staining [17]. Osmium tetroxide electronically stains osmiophilic compounds in native tissues and malachite green molecules bound to tissues. Imidazole promotes the reaction of osmium tetroxide. *p*-Phenylenediamine reduces osmium tetroxide bound to cellular materials and increases electron density [18]. The results of the spot test showed that the MGIP method is also suitable for observing standard lipids such as fatty acids and triglycerides in plant and animal cells when the lipid has carbon double bonds.

However, undulation of the plasma membrane and plasmolysis, which likely represent artifacts caused by chemical fixation, were observed in most samples of M9 cultured cells even though the cells were fixed by the MGIP method. Thus, the freeze-substitution method may be required to observe the native shape of subcellular structures, especially the plasma membrane.

### Metabolism of lipophilic shikonin derivatives in *L. erythrorhizon* cells

Shikonin derivatives are highly lipophilic secondary metabolites produced by *L. erythrorhizon*, and the production rate reaches 10% (w/w) of the cell dry weight [6]. High-electron-dense lipid droplets were often observed in shikonin-producing cells cultured in M9 medium. In contrast, only a few lipid droplets were observed in shikonin-non-producing cells cultured in LS medium. This indicates that these lipid droplets are shikonin derivatives or their precursors. The cells cultured in M9 medium showed several characteristic cellular structures in addition to the high production of lipid droplets. A highly developed and fragmented ER was often observed, especially in cells secreting shikonin to the extracellular spaces, where it accumulated as shikonin granules attached to the cell wall (Fig. 4c, d) and was partly suspended in the medium [3,26]. This observation indicates that the ER plays an important role in shikonin biosynthesis and its secretion, as reported previously [7,8,38].

Cells cultured in M9 medium showed more intense undulation of the plasma membrane than those cultured in LS medium (Figs. 4 and 6). In addition, some of the cells secreting shikonin showed folded plasma membranes and tonoplasts (Fig. 5a and b). Such folded structures are called mesosomes and have been reported to be an artifact caused by chemical fixation [39,40]. However, lipid droplets were sometimes observed just outside the undulated or folded plasma membranes (Figs. 5b, arrowhead, 7b, 8b). This observation indicates that these undulations and foldings may represent movements of the plasma membrane for exporting lipophilic metabolites to extracellular spaces. Investigating the possible artifactual nature (or not) of these structures represents future research with the freeze-substitution method. Folded tonoplasts were also observed (Fig. 5d, arrows), and high-electron-dense materials were attached to tonoplasts (Fig. 5d, asterisks). These structures appear to be membrane fusion of the compounds related to shikonin production. Yazaki *et al*. [41] reported that *p-O-β*-d-glucosylbenzoic acid, a precursor of shikonin derivatives, is stored in vacuoles in shikonin-non-producing cells until utilized as a precursor upon induction of shikonin biosynthesis [42]. This past report and TEM observations in this report suggest that the shikonin precursor is transported within a lipid membrane structure to the tonoplast. Membrane fusion then occurs at the tonoplast to facilitate the transport of the precursor to the cytoplasm. In addition, the precursor is water-soluble and must have been extracted during prefixation. Therefore, the presence of high-electron-dense material indicates that the precursor is converted or bound to osmiophilic compounds at the same place of membrane fusion [43,44]. Another possibility of the electron-dense materials attached to the tonoplast is phenolic substances actively produced in M9 medium, such as lithospermic acid B, which is a caffeic acid tetramer and accumulated inside the cells contrary to shikonin derivatives [3,26]. Adding to rosmarinic acid, a caffeic acid dimer, these phenolic substances are representative tannin-like phenolics in Boraginaceae and Lamiaceae [45].

### Effect of post-embedded staining on samples fixed by the MGIP method

In the MGIP method, cellular structures were sufficiently visible except for cell walls, even though post-embedded staining was omitted (Fig. 7). The observed low electron density of cell walls is because of the absence or low concentration of the osmiophilic or malachite-green-affinity materials in callus cell walls. Double staining with uranyl acetate and lead citrate electronically stained the cell wall and lipid droplets and improved the clarity of organelles (Figs. 4 and 5). However, potassium permanganate staining showed higher electron staining effects than double fixation (Figs. 4, 5 and 8). This is unexpected because Mn is a lighter atom than U and Pb. Some fixative reagents may react with potassium permanganate, but we cannot explain the detailed mechanism.

## Concluding remarks

Compared with conventional chemical fixation methods, fixation and visualization of lipid droplets and organelles were improved when fixation with glutaraldehyde containing malachite green was followed by imidazole–osmium and *p*-phenylenediamine treatments (MGIP method). In cultured *L. erythrorhizon* cells, lipid droplets showed high electron density, and subcellular structures were clearly visible under TEM. TEM observation of the cultured cells fixed by the MGIP method indicated that production and secretion of shikonin derivatives involve complex development of the ER and folding of the plasma membrane, and *p-O-β*-d-glucosylbenzoic acid, a water-soluble shikonin precursor, is transported to the cytosol and converted or bound to osmiophilic compounds at the same place of membrane fusion.

## Supplementary Material

dfac018_Supp

## Data Availability

Data sharing is not applicable to this article.
